# Predictors of health-related quality of life changes after lifestyle intervention in persons at risk of type 2 diabetes mellitus

**DOI:** 10.1007/s11136-014-0702-z

**Published:** 2014-05-01

**Authors:** Vegard Nilsen, Per Sigvald Bakke, Gudrun Rohde, Frode Gallefoss

**Affiliations:** Medical Department, Sorlandet Hospital Kristiansand, Service box 416, 4604 Kristiansand, Norway

**Keywords:** Quality of life, Type 2 diabetes mellitus, Prevention, Lifestyle, Obesity

## Abstract

**Purpose:**

To assess health-related quality of life (HRQOL) of subjects at risk of type 2 diabetes undergoing lifestyle intervention, and predictors for improved HRQOL.

**Methods:**

The Finnish Diabetes Risk Score was used by general practitioners to identify individuals at risk. Low-intensity interventions with an 18-month follow-up were employed. HRQOL was assessed using the SF-36 at baseline and compared with results from a general Norwegian population survey and further at 6 and 18 months. Simple and multiple linear regression analyses were applied to identify predictors of changes in HRQOL of clinical importance.

**Results:**

Two hundred and thirteen participants (50 % women; mean age: 46 years, mean body mass index: 37) were included: 182 returned for 18-month follow-up, of whom 172 completed the HRQOL questionnaire. HRQOL was reduced with clinical significance compared with general Norwegians. The mean changes in HRQOL from the baseline to the follow-up were not of clinical importance. However, one out of three individuals achieved a moderate or large clinical improvement in HRQOL. The best determinant for improved HRQOL was obtained for a composite, clinically significant lifestyle change, i.e. both a weight reduction of at least 5 % and an improvement in exercise capacity of at least 10 %, which was associated with an improvement in five out of the eight SF-36 domains.

**Conclusion:**

Subjects at risk of type 2 diabetes report a clinically important reduction in HRQOL compared with general Norwegians. The best predictor of improved HRQOL was a small weight loss combined with a small improvement in aerobic capacity.

## Introduction

Lifestyle modification in subjects at high risk of type 2 diabetes mellitus (DM) has been proven effective in reducing the incidence of type 2 DM [1–3]. Two systematic reviews that assessed the effects of lifestyle changes on the prevention of type 2 DM showed that no studies reported data on health-related quality of life (HRQOL) [4, 5]. The negative consequences of both type 2 DM and obesity on HRQOL have been well documented [6–8]. Significant HRQOL improvements have been observed after weight loss in obese individuals undergoing a variety of treatments [7, 9], although a systematic review of randomised trials reported inconsistent results [10]. The relative importance of weight loss versus improved fitness regarding the improvement in HRQOL via lifestyle modification is unclear. Among women, weight loss seems to be the main contributor to improved HRQOL, whereas increased fitness yielded disappointing effects [11]. In the Diabetes Prevention Program, all facets of the significant improvement in HRQOL observed were correlated primarily with weight loss [12].

The aim of this study was to assess HRQOL in an unselected group of subjects at risk of type 2 DM undergoing lifestyle treatment and to identify predictors of clinically important HRQOL improvements. Low-intensity interventions with high applicability in ordinary clinical practice were chosen.

## Methods

### Subjects and study design

Individuals at high risk of type 2 DM were identified by general practitioners (GPs) using the seven-item “Finnish Diabetes Risk Score” (FINDRISC) [13]. FINDRISC is based on traditional risk factors for diabetes, such as body mass index (BMI), waist circumference, inactivity and age. Copies of the FINDRISC questionnaire were sent by post to approximately 90 GPs in the four municipalities located nearest to the hospital. Individuals aged 18–64 years with a FINDRISC score ≥9, which implies a moderate-to-high risk of type 2 DM, were invited to participate in the study. Study inclusion was performed from March 2004 to September 2005, with an 18-month follow-up period. After signing written informed consent, participants were allocated randomly to an “individual physician group” (IG) or an “individual physician plus interdisciplinary group” (IIG). Individual physician interventions in both groups were delivered at baseline and at 6, 12 and 18 months. Subjects in the IIG participated in an additional 18 h group-based, interdisciplinary programme administered over 16 weeks. Since no statistically significant differences between intervention groups were found regarding change in lifestyle or change in HRQOL, the results are presented as a cohort study for all participants combined [14]. Details regarding recruitment methods, FINDRISC, exclusion criteria, the intervention programme and categorisation of aerobic capacity and diet have been thoroughly explained previously [14]. The study was approved by the Regional Committee for Medical Research Ethics of southern Norway.

### Assessments

Socio-demographic features, height without shoes, weight in indoor clothes and the results of a modified Bruce protocol on a treadmill for subjects with low aerobic capacity were recorded at baseline and again after 6 and 18 months, yielding maximal oxygen uptake reported as mL/kg/min. A weight reduction ≥5 % and an improvement in exercise capacity of ≥10 % from the baseline to the follow-up were used as criteria for a clinically significant lifestyle change [14]. HRQOL was assessed at the baseline, 6 and 18 months using the Medical Outcomes Survey, Short Form 36 (SF-36), version 1. This is a generic instrument that has been extensively tested nationally and internationally and has satisfactory reliability and validity. The SF-36 has proven to be applicable to both healthy subjects and patients with medical conditions, thereby rendering it possible to draw comparisons between patients and the general population [15, 16]. Normative data from the general Norwegian population (*n* = 4,444) were used for comparison [17]. The answers to the 36 items are coded into eight domains; four are interpreted as physical indicators (general health perception (GH), physical functioning (PF), role limitation physical (RP) and bodily pain (BP)) and four are interpreted as mental health indicators (mental health (MH), social functioning (SF), vitality (VT) and role limitation emotional (RE)). The eight domains are transformed to a scale of 0 to 100, in which 100 is the best possible and 0 the worst possible health state [15]. Norwegian SF-36 norm data for the age-group were used to aggregate the two summary scales from *z*-score transformations of the eight domains, a physical component summary (PCS) and a mental component summary (MCS) [16]. These summary scales are standardised, to achieve a mean score of 50 and a standard deviation of 10 in the general population. Scores above 50 represent better functioning compared with the general population and vice versa.

### Definition of end-points

One of the challenges of studying HRQOL is that improvements that are statistically significant can, nevertheless, be of little clinical relevance [18]. The primary outcomes of this paper were clinically important changes in HRQOL. On a 100-point scale, mean score changes of 5–10 points were interpreted as small, changes of 10–20 points were considered moderate and changes of >20 points were considered large clinical changes [19, 20]. Regarding the summary scales (PCS and MCS), a 2–5 point change was interpreted as small, a 5–8 point change was considered moderate and a ≥8 point change was considered large, corresponding to effect sizes of 0.20–0.49, 0.50–0.79 and ≥0.80 [16, 20]. Changes (*Δ* values) in the eight domains and two summary scores were calculated by subtracting the baseline value from the follow-up value, i.e. a positive value implies an improvement, whereas a negative value implies a worsening of HRQOL.

### Statistical analyses

Statistical analyses were carried out using the Statistical Package for Social Sciences (SPSS), version 18.0. Differences in means between groups were assessed using an independent samples *t* test for continuous variables with normal distribution, and the *χ* ^2^ test was used for categorical variables. Mean differences between the study group and normative data were assessed using the *t* test. Paired sample *t* test was used to detect changes in HRQOL data over time.

Simple linear regression analyses and multiple linear regression analyses (GLM procedure in SPSS) were applied to identify significant predictors of changes in HRQOL from baseline to follow-up for the eight domains and the two summary scales, with adjustment for baseline HRQOL values in the multiple analyses. Independent variables in the multiple regression analyses were selected based on both clinical experience and findings from a previous study that showed that socio-demographic variables (age, sex, living conditions and education) influence HRQOL [21]. Further, regarding the uncertainty about the relative importance of weight loss versus improved fitness regarding the improvement in HRQOL, the weight goal alone, the aerobic capacity goal alone and the two combined were tested in the multiple linear regression analyses. To strengthen the analyses for the combined lifestyle achievement, multiple logistic regression analyses were also performed using the same independent variables; these yielded the odds ratios (ORs) for at least a small, clinically significant change in HRQOL as the dependent variable. Confidence intervals (CIs) were reported at the 95 % level. The level of significance was set at *p* ≤ 0.05.

## Results

**Fig. 1:**
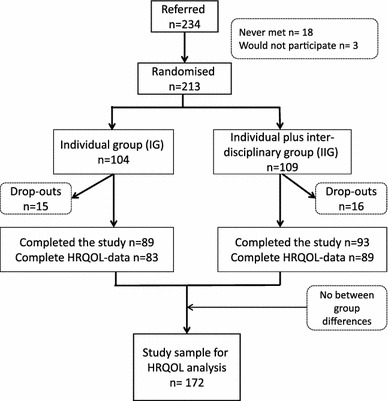
Flow diagram of participant selection throughout the trial

Sixty-five of the ~90 GPs who received the FINDRISC questionnaires referred at least one subject from March 2004 to September 2005. Out of the 234 eligible subjects at risk, all 213 individuals who wanted to participate were included in the study (Fig. 1). Of the 213 randomised subjects, 212 completed the SF-36 questionnaire at baseline and 172 (81 % of the randomised individuals) of the 182 subjects who attended the follow-up assessment completed the final SF-36 questionnaire. Unhealthy lifestyle parameters were prevalent: The mean BMI was 37, 90 % of subjects had a BMI >30, three out of five had an unhealthy diet, more than 50 % had poor aerobic capacity, and every fourth participant smoked daily (Table 1). Compared with the general Norwegian population, the population at risk of type 2 DM reported at baseline both statistically significant and clinically important deficits in HRQOL for all eight domains of the SF-36 and for the summary scores (Table 2), with the greatest disparities observed for the physical domains. The 15 % of subjects who dropped out reported clinically important deficits in HRQOL scores at baseline than did the completers of the study (Table 2).

**Table 1:** Baseline characteristics. Values are means with standard deviation (SD) or percentage

	All
	*n* = 213
Socio-demographic data	
Age	46.5 (11)
Gender, men (%)	50
Married or cohabiting, %	74
High school/university, %	28
Employed, %	62
Long-term sick leave/disabled, %	32
Daily smoker, %	25
Weight measures	
Weight, kg	112.2 (22)
Body mass index, kg/m^2^	36.8 (6.0)
Waist circumference, cm	119 (14)
Diet	
Healthy diet, %	1
Somewhat unhealthy diet, %	39
Unhealthy diet, %	60
Aerobic capacity, ml/kg/min (*n* = 201)	26.8 (7.6)
Good, excellent or superior aerobic capacity, %	25
Poor or very poor aerobic capacity, %	55

**Table 2:** Baseline values for HRQOL (SF-36) in the study population, from the general Norwegian population and from completers versus dropouts of the study

SF-36 domain*	All	Norm #	Completers	Dropouts
	*n* = 212	*n* = 4,444	*n* = 182	*n* = 30
Bodily pain (BP)	60 (29)	75 (25)***	62 (28)	46 (31)**
General health (GH)	58 (24)	77 (21)***	60 (24)	42 (22)***
Physical function (PF)	75 (20)	90 (17)***	77 (19)	63 (21)***
Physical role limitation (RP)	64 (41)	82 (34)***	66 (40)	49 (42)*
Mental health (MH)	74 (18)	80 (15)***	76 (17)	64 (23)**
Social function (SF)	79 (26)	87 (20)***	81 (24)	65 (31)**
Vitality (VT)	47 (23)	61 (20)***	49 (22)	37 (23)**
Emotional role limitation (RE)	76 (37)	87 (29)***	79 (36)	55 (41)**
Physical component summary (PCS)	41 (12)	50 (10)***	42 (12)	36 (11)*
Mental component summary (MCS)	47 (13)	50 (10)***	48 (12)	41 (15)*

Data are presented as means (SD). *Norm #* normative data from the general Norwegian population aged 18–64 years. Independent samples *t* test between the whole study population (All) and Norm **#** and Completers and dropouts

* *p* < 0.05, ** *p* < 0.01 and *** *p* < 0.001)

The mean weight loss and mean increase in maximal aerobic capacity from the baseline to the follow-up were 2 % (SD, 6) and 12 % (SD, 25), respectively. Correspondingly, the mean changes in all HRQOL scores were small and not of clinical importance (Table 3, 4). However, a moderate or large clinical improvement in HRQOL was achieved in about one out of three participants, with the highest proportions found for general health (42 %) and vitality (41 %), the lowest for emotional role limitation (18 %) and the two summary scales in the middle with PCS (32 %) and MCS (31 %). The improvements in HRQOL were basically achieved during the first 6 months and thereafter stabilised (Table 4).

**Table 3:** *Δ* values from the baseline to the follow-up for HRQOL (SF-36) shown for all subjects and for those achieving and not achieving two main lifestyle change goals (weight reduction ≥5 % and improved aerobic capacity ≥10 %)

SF-36 domain*	All	Not achieving both goals	Achieving both goals
	*n* = 172	*n* = 96	*n* = 26
Bodily pain (BP)	0 (25)	−2 (22)	13 (29)*
General health (GH)	4 (18)	2 (17)	14 (16)**
Physical function (PF)	5 (16)	5 (13)	17 (15)***
Physical role limitation (RP)	0 (40)	1 (37)	15 (42)
Mental health (MH)	2 (18)	2 (16)	7 (16)
Social function (SF)	3 (25)	0 (21)	14 (24)**
Vitality (VT)	5 (22)	3 (22)	17 (16)**
Emotional role limitation (RE)	3 (39)	6 (30)	3 (38)
Physical component summary (PCS)	2 (9)	1 (8)	8 (9)***
Mental component summary (MCS)	1 (13)	1 (11)	4 (11)

Data are presented as means with standard deviations in parenthesis. *p* values based on an independent samples *t* test performed between those achieving and not achieving the lifestyle change (* *p* < 0.05, ** *p* < 0.01 and *** *p* < 0.001)

**Table 4:** Mean HRQOL values (SF-36) from baseline and 6 months to 6 and 18-month follow-up, respectively

SF-36 domain*	Baseline	6 months	6 months	18 months	Baseline	18 months
	*n* = 166	*n* = 166	*n* = 150	*n* = 150	*n* = 172	*n* = 172
Bodily pain (BP)	62 (27)	62 (27)	63 (27)	62 (29)	62 (28)	62 (30)
General health (GH)	**60 (23)**	64 (23)*	64 (23)	64 (23)	**59 (24)**	64 (23)**
Physical function (PF)	**78 (18)**	80 (18)**	81 (17)	82 (17)	**76 (19)**	81 (18)***
Physical role limitation (RP)	**65 (40)**	71 (38)*	**73 (37)**	65 (40)**	65 (40)	65 (41)
Mental health (MH)	76 (17)	77 (17)	78 (16)	77 (18)	76 (17)	78 (17)
Social function (SF)	81 (25)	84 (23)	84 (22)	83 (23)	80 (25)	83 (23)
Vitality (VT)	**49 (21)**	53 (22)*	53 (22)	52 (24)	**48 (22)**	53 (23)**
Emotional role limitation (RE)	79 (36)	84 (31)	85 (30)	81 (35)	79 (36)	81 (34)
Physical component summary (PCS)	**42 (11)**	44 (11)*	44 (11)	44 (11)	**42 (12)**	44 (12)*
Mental component summary (MCS)	48 (13)	50 (12)	50 (12)	49 (14)	48 (13)	50 (13)

Paired sample *t* test. Data are presented as means with standard deviations in parentheses. Values marked with bold indicate statistical significance (* *p* < 0.05, ** *p* < 0.01 and *** *p* < 0.001)

A simple linear regression analysis uncovered that improved PCS was correlated with weight loss and improved fitness, respectively, i.e. 1.5 points for every 5 kg lost and 3.4 points for every 5 mL/kg/min improvement in maximal aerobic capacity. No significant correlations were identified for improved MCS.

**Fig. 2:**
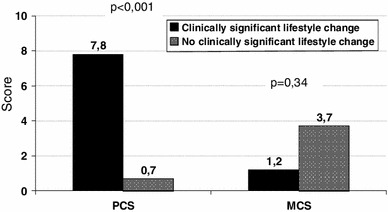
Changes in PCS and MCS scores associated with achieving or not achieving the combined lifestyle change (weight reduction ≥5 % and improvement in exercise capacity ≥10 % from the baseline to the follow-up) based on independent *t* tests

In a multiple linear regression analysis, using HRQOL score as the dependent variable revealed that a weight reduction ≥5 % alone was associated with improvement in one physical domain (GH with *B* = 7.6 (2.4–12.9)), one mental domain (VT with *B* = 8.4 (2.3–14.5)) and one summary scale (PCS with *B* = 2.9 (0.2–5.6). In the same model, an improvement in exercise capacity ≥10 % alone was correlated with improvement in only one physical domain (BP with *B* = 8.9 (0.8–17.1)) and one summary scale (PCS with *B* = 3.5 (0.6–6.5). Further, this model demonstrated that the best predictor of improved HRQOL was a clinically significant lifestyle change defined as both a weight reduction ≥5 % and an improvement in exercise capacity ≥10 % from the baseline to the follow-up (Table 5). This combined lifestyle change was associated with improvement in three of four physical domains (not RP), two out of four mental components (VT and SF) and one of the two summary scales (PCS) of the SF-36 questionnaire (Table 5). The achievement of this composite lifestyle change was correlated with a large effect on PCS score compared with individuals who did not achieve it, with an unadjusted improvement on PCS of 7.8 (3.4–10.7) and an adjusted improvement of 6.4 (2.9–9.8) (Fig. 2; Table 5).

**Table 5:** Predictors of improvement in health-related quality of life (SF-36) in a multiple linear regression analyses with adjusted regression coefficients (adj B)

	Bodily pain (BP) Adj B (95 % CI)	*p*	General health (GH) Adj B (95 % CI)	*p*	Physical function (PF) Adj B (95 % CI)	*p*	Physical role (RP) lim. Adj B (95 % CI)	*p*	Mental health (MH) Adj B (95 % CI)	*p*
Demographics										
Male	5.2 (−2.5–12.9)	0.19	0.5 (−5.3–6.3)	0.86	−0.4 (−4.4–3.7)	0.85	4.6 (−7.0–16.2)	0.43	2.3 (−3.0–7.7)	0.39
High education	5.0 (−3.5–13.5)	0.24	0.7 (−5.7–7.2)	0.82	0.7 (−3.7–5.2)	0.75	−7.4 (−20.2–5.3)	0.25	−4.8 (−10.6–1.1)	0.11
Working at baseline	7.9 (−1.0–16.8)	0.08	3.9 (−2.6–10.5)	0.24	**5.1 (0.4–9.8)**	**0.03**	**24.3 (10.5**–**38.2)**	**0.001**	0.7 (−5.2–6.6)	0.82
Living together	1.3 (−7.7–10.3)	0.78	−2.8 (−9.5–4.0)	0.42	0.6 (−4.2–5.5)	0.80	−2.2 (−15.8–11.4)	0.75	0.4 (−5.8–6.6)	0.91
Age*	0.1 (−0.3–0.5)	0.57	0.1 (−0.2–0.4)	0.61	0.0 (−0.2–0.2)	0.76	0.2 (−0.4–0.8)	0.45	0.1 (−0.2–0.3)	0.65
IIG group	0.1 (−7.8–8.0)	0.57	0.7 (−5.1–6.6)	0.81	−0.2 (−4.3–3.9)	0.93	−4.7 (−16.4–7.1)	0.43	−2.6 (−8.0–2.7)	0.33
Improved weight and fitness**	**15.4 (5.7–25.1)**	**0.002**	**12.7 (5.4–19.9)**	**0.001**	**8.9 (3.8–14.0)**	**0.001**	12.1 (−2.4–26.6)	0.10	5.6 (−1.1–12.2)	0.10
Adjustment for baseline value***	−0.4 (−0.5–0.2)	<0.001	−0.3 (−0.5–0.2)	<0.001	−0.5 (−0.6–0.3)	<0.001	−0.6 (−0.8–0.4)	<0.001	−0.5 (−0.7–0.3)	<0.001
*R* ^2^ adjusted	22.3 %		19.1 %		39.1 %		32.2 %		20.6 %	

	Social function (SF) Adj B (95 % CI)	*p*	Vitality (VT) Adj B (95 % CI)	*p*	Emot. role (RE) lim. Adj B (95 % CI)	*p*	Phys. comp. sum. (PCS) Adj B (95 % CI)	*p*	Ment. comp. sum (MCS) Adj B (95 % CI)	*p*
Demographics										
Male	1.7 (−5.0–8.5)	0.61	3.0 (−4.0–10.0)	0.40	3.0 (−7.0–12.9)	0.56	1.1 (−1.6–3.8)	0.44	0.7 (−3.3–4.7)	0.74
High education	−5.5 (−12.9–1.9)	0.15	−2.5 (−10.1–5.1)	0.52	−3.2 (−14.0–7.6)	0.56	0.8 (−2.2–3.7)	0.62	−2.9 (−7.2–1.5)	0.19
Working at baseline	**8.1 (0.6–15.5)**	**0.04**	5.6 (−2.1–13.4)	0.15	−4.6 (−15.5–6.3)	0.41	**4.3 (1.1–7.5)**	**0.009**	−1.1 (−5.4–3.2)	0.61
Living together	3.3 (−4.5–11.2)	0.40	−1.5 (−9.5–6.5)	0.71	−4.7 (−16.2–6.7)	0.41	−0.8 (−3.7–2.2)	0.62	−0.1 (−4.6–4.5)	0.98
Age*	0.3 (−0.1–0.6)	0.13	0.0 (−0.3–0.4)	0.88	−0.1 (−0.6–0.4)	0.70	0.0 (−0.1–0.2)	0.48	0.0 (−0.2–0.2)	0.94
IIG group	−2.4 (−9.2–4.4)	0.49	−5.8 (−12.7–1.1)	0.10	−4.6 (−14.6–5.4)	0.36	0.2 (−2.6–3.0)	0.89	−2.7 (−6.7–1.3)	0.18
Improved weight and fitness**	**11.3 (2.9–19.7)**	**0.01**	**11.3 (2.8–19.9)**	**0.01**	−1.3 (−13.6–11.1)	0.84	**6.4 (2.9–9.8)**	**<0.001**	2.2 (−2.7–7.1)	0.38
Adjustment for baseline value***	−0.5 (−0.7–0.4)	<0.001	−0.4 (−0.6–0.3)	<0.001	−0.5 (−0.7–0.4)	<0.001	−0.4 (−0.5–0.3)	<0.001	−0.4 (−0.6–0.2)	<0.001
*R* ^2^ adjusted	31.5 %		22.2 %		31.6 %		28.7 %		15.6 %	

Regression analyses of demographics, clinical characteristics and baseline values for the eight domains for HRQOL and the two summary scales. Each independent variable was compared with its “opposite”, i.e. females with males, low with high education, no work with work, etc. *p* values marked with bold indicate statistical significance. *R* ^2^ represents how well the model explains the dependent variable. * Age in years. ** Both a weight reduction ≥5 % and an improvement in exercise capacity of ≥10 % from the baseline. *** Influence of the baseline value of each domain on the delta value

Based on a multiple logistic regression model, the adjusted ORs for small clinically significant improvements in HRQOL for achievers of the composite lifestyle change versus non-achievers were statistically significant for three physical domains (GH, PF and BP), but none of the mental domains or the summary scales. The OR was highest for GH (7.0 (2.2–21.8)) and quite similar for PF (3.9 (1.2–13.3)) and BP (4.0 (1.4–12.1)).

## Discussion

This study showed that subjects at risk of type 2 DM had markedly lower HRQOL than did the general Norwegian population on all eight domains of the SF-36 and on the PCS and MCS summary scales. However, HRQOL improvement in clinical importance was accomplished by a moderate lifestyle change achieved with modest clinical efforts.

### The limitations of the study must be considered

First, dropouts differed from completers of the study, reporting significant decrements in HRQOL at the baseline. Thus, individuals who were most dissatisfied with their lives and who were in most need of a lifestyle change unfortunately seemed to dropout of the study. This observation coincided with results from a large meta-analysis and the experiences of many health care providers: “Those who need it the most, understand it the least”, which represents a major healthcare challenge [22].

Second, HRQOL was assessed using a generic instrument, not a disease-specific one. An obesity-specific instrument may have better sensitivity to detect changes than a generic one. The generic SF-36 was chosen since we only included subjects at risk of a disease. A mean BMI of 37 was a surprising finding in this study. Conversely, one of the major advantages of a generic questionnaire is the possibility to draw comparisons between the study group and the general population and between a variety of medical conditions [6].

Third, the results of this study may be biased by a clustering bias of GPs referring the patients to the study or a selection bias through the participants’ willingness to participate. However, we are not, the way the study was designed, able to correct for these biases. Further, the attendance rate at the final fitness test weakens the study results assessing predictors, also due to a possibility of selection bias, i.e. those who achieve lifestyle changes turn up for the final assessment to a larger extent than those who do not.

Fourth, regarding the applicability of the results, the effects may have been overestimated because of a healthy volunteer bias: Volunteers are fitter and healthier than non-volunteers [23, 24]. On the other hand, as shown in Table 5, baseline values for all ten variables from the SF-36 questionnaire are inversely correlated with improvements in the same variables, i.e. HRQOL seems easier to improve if baseline values are low compared to high, thereby supporting the general tendency of the “regression to the mean” bias. This may support a tendency towards underestimation of the effects if those with even lower HRQOL had participated in the study. However, we are not able to exploit these potential biases thoroughly.

Finally, a follow-up time of 18 months does not automatically imply that the effects achieved are sustainable. It is common knowledge in lifestyle interventions weight loss studies that results diminish overtime [22]. We have no further follow-up assessment data.

The strengths of this study were as follows: First, the simple selection of eligible patients by GPs using the FINDRISC questionnaire. Second, an inclusion rate >91 %, a participation rate >98 %, the absence of excluded subjects and a dropout rate ≤15 % are all robust characteristics for this clinical study [14]. The general applicability of these results to common clinical settings should thus be good.

As 90 % of the study population was obese, and obesity is related to a lower HRQOL [6], the finding of reduced HRQOL in this study was as expected; however, the magnitude of the difference compared with the general Norwegian population was surprising. Two large meta-analyses have shown that, among obese persons, those not seeking treatment have the best HRQOL, those seeking conservative treatment have a more moderate HRQOL and those seeking surgery have the worst HRQOL [25, 26]. It is surprising that the subjects in this study, who were not seeking treatment for obesity, but were assessed to be at risk of type 2 DM through a questionnaire survey, reported an HRQOL that was as low as that of subjects undergoing bariatric surgery [26]. Decreased HRQOL in subjects at risk of type 2 DM is not a new finding [27, 28]. However, in contrast to findings from Finland, where subjects at risk reported lower general health and increased bodily pain compared with the general Finnish population, all eight dimensions of the SF-36 were significantly lower in our study [28]. This can be explained by the much higher prevalence of obesity in the present study (90 %) compared with the study from Finland (31 %). Chittleborough et al. studied HRQOL along the diabetes continuum in Australia and found a significantly lower score for bodily pain exclusively (i.e. increased pain) among those with impaired fasting glucose compared with those with normal glucose levels, whereas those with diabetes scored significantly lower on all dimensions, with the exception of mental health [27].

The relative importance of weight loss versus improved fitness regarding the improvement in HRQOL in this study showed an improvement of 1.5 PCS points for every 5 kg lost and 3.4 points for every 5 mL of improvement in maximal aerobic capacity (mL of O_2_ uptake/kg/min). No correlations between changes in body weight or fitness and MCS were found. Correspondingly, improvements in only two of the eight and one of the eight domains of the SF-36 were associated with weight loss or fitness improvement alone, respectively. However, the combination of both weight reduction and improved fitness was most highly correlated with improved HRQOL. Five out of the eight domains of the SF-36 were significantly improved in subjects who made a clinically significant lifestyle change (Table 5). Nevertheless, the correlation observed for **Δ**PCS was very weak, with an adjusted *R* ^2^ of 0.287, which means that only 28.7 % of the variation observed can be explained by this lifestyle change. In other words, most of the variation in **Δ**PCS could not be explained by the variables identified in this study. However, the study showed that subjects who attained clinically significant lifestyle changes exhibited an improved HRQOL. The greatest impact was found for physical HRQOL domains of functioning, which was in accordance with the results of other studies [6]. Subjects who exhibited an improvement in both weight and fitness may experience a new way of living when approximating their motivational goals. Our experience is that those who achieve both weight reduction and improved fitness often become very dedicated to their changes in lifestyle, in a way that is very similar to that adopted by those who want to quit smoking or alcohol abuse. Achieving their goals after such large motivational changes can then lead to a considerable improvement in reported HRQOL.

A large meta-analysis has shown that an obesity-specific HRQOL instrument reflected weight-related QOL with much better sensitivity than did the SF-36, and found that factors other than weight change were crucial for HRQOL changes [26]. The finding of a much lower HRQOL in the subjects included in this study compared with the general population based on the generic instrument SF-36 may be due to more emotional and complex problems in life, for which weight loss is not the “simple” solution. Obesity is a major public health problem, as a risk factor for a variety of illnesses and as having a devastating impact on HRQOL. This study confirmed the negative consequences of obesity on HRQOL. It also confirmed that even small changes in lifestyle may enhance HRQOL significantly, and that most subjects at risk of type 2 DM are obese, which are all in accordance with the findings of other reviews [6, 7]. Many health care professionals argue that, regarding obesity, for which a cure is unlikely, one of the most important health outcomes that warrants evaluation and improvement is quality of life [7]. We believe that lifestyle changes at a moderate level, as exemplified by a modest increase in physical activity and a small weight loss, will be the most important elements in improving HRQOL for subjects at risk of type 2 DM. Improvement in HRQOL should, perhaps, be the main goal at the start of treatment, as this may increase the chances for further therapeutic success. In the future, preventive programmes including weight control and exercise should be established for the large proportion of subjects at risk of type 2 diabetes. An individual approach, such as that shown in this study, can be used with modest clinical efforts while yielding clinically important results.

## Conclusions

In summary, this study of subjects at risk of type 2 DM showed that HRQOL was markedly reduced in this population. A clinically important improvement in HRQOL was clearly correlated with the achievement of a composite lifestyle change (weight reduction and improved aerobic capacity). However, correlation is not causation. But this association may indicate that important HRQOL improvements can be achieved by small improvements in lifestyle changes in subjects at risk of type 2 diabetes.
